# A Scoping Review of Mental Health Needs and Challenges among Medical Students within South African Universities

**DOI:** 10.3390/ijerph21050593

**Published:** 2024-05-04

**Authors:** Mokhwelepa Leshata Winter, Sumbane Gsakani Olivia

**Affiliations:** School of Medicine, Faculty of Health Science, University of Limpopo, Private Bag X 1106, Sovenga 0727, South Africa; gsakani.sumbane@ul.ac.za

**Keywords:** medical students, mental health, challenges, South Africa, universities, needs, support systems, well-being

## Abstract

The mental health of medical students is a growing concern worldwide, with studies indicating high levels of stress, anxiety, and depression among this population. In a South African context, this review aims to review the existing literature on mental health needs and challenges among medical students in South Africa. The rationale for this review is crucial to identify gaps, understand unique contextual factors, and inform the development of targeted interventions and support tailored to the specific needs of South African medical students. This review followed a scoping review framework by Arksey and O’Malley which consists of five stages. The review was initiated in December 2023. The search process was conducted on the following electronic databases: PubMed, Psych-info, Scopus, Google Scholar, and Science Direct. The search terms of this review were “Medical students” OR “Mental health”, OR “Challenges”, OR “South Africa”, OR “Universities” OR “Needs”, OR “Support systems”, OR “Mental health interventions”. This study included articles published in English between 2010 and 2023. After a thorough review of the literature, only eight articles met the inclusion criteria. This study excluded articles that were not published in English, articles published before 2010, full-text articles that could not be retrieved, and studies that did not address the mental health needs and challenges faced by medical students and risk factors contributing to mental issues among South African medical students. The review yielded only three themes utilizing Creswell’s Tesch method of data analysis. (1) Prevalence of mental health disorders, (2) risk factors contributing to poor mental health, and (3) available university support systems and interventions. Therefore, the unique aspect of our review lies in shedding light on the underexplored intersections between mental health and the unique context of medical education in South Africa. This includes examining the impact of historical, cultural, and institutional factors on the mental health and well-being of medical students, which has not been comprehensively addressed in previous literature in terms of the South African context. The findings of this review highlight the importance of implementing comprehensive mental health support programs within medical education institutions to address the needs of students and promote their well-being.

## 1. Introduction

Global attention to medical students’ mental health and wellness has increased recently, indicating a growing understanding of the particular stresses and difficulties they experience [1]. The University of Cape Town, University of Stellenbosch, University of Free-State, University of KwaZulu-Natal, University of Pretoria, University of the Witwatersrand, Walter Sisulu University, Nelson Mandela University, and University of Limpopo are ten of the medical schools that are part of South African universities. But according to the World Health Organization (WHO), mental health issues have a major role in morbidity and disability [2].

According to systematic evaluations, medical students have higher rates of depression or depressive symptoms (27.2%) than their age-matched counterparts, indicating a disproportionate impact on their mental health [3]. Between the ages of 18 and 35, when students receive training, according to the South African Stress and Mental Health (SASH) survey, the prevalence of major depressive disorder (MDD) is 8.9% and that of anxiety-related disorders is 14.6% among students who typically receive training [4]. Data on medical students in South Africa (SA) are, however, scarce [4]. Undergraduate medical students in South Africa receive extensive and demanding training. In South Africa, medical training typically begins at the undergraduate level [5]. The training encompasses both theoretical learning and practical clinical experience, often characterized by rigorous coursework and demanding clinical rotations [5]. Regarding depression and anxiety outcomes, there could indeed be variations between undergraduate medical students and those attending secondary medical schools. Factors such as the intensity of academic pressure, exposure to clinical environments, and support systems available may differ between these groups, potentially impacting mental health outcomes [5]. These factors have been found to perpetuate the rates of mental illness among South African medical students. It is very important to note that further research comparing these cohorts could provide valuable insights into the unique challenges they face. Financial hardships, heightened competition, cultural and linguistic isolation, and inadequate preparation for postsecondary education are examples of contextual issues that impact their experience [4]. Additionally, there is a greater chance of mental illness susceptibility due to the accumulation of distinct sociopolitical exposures, which might be felt as long-term stresses [6,7,8]. Pressure on students to bring their communities out of poverty, frequent exposure to social injustice, victimization and violence, dangers to one’s safety, a delay in institutional change, and the effect of student demonstrations on mental health are a few examples of these [4].

The impact of COVID-19 on mental health outcomes among medical students in South Africa likely varied and could have been significant [9]. The pandemic introduced numerous stressors, including increased workload, changes in learning environments, fear of infection, isolation due to lockdown measures, and uncertainty about the future [9]. Medical students faced additional challenges, such as disrupted clinical rotations, concerns about their own health and that of their families, and ethical dilemmas related to patient care during the pandemic [9]. However, many medical schools in South Africa do offer counseling services specifically tailored to their students’ needs [4], for example, the University of Pretoria, University of Cape Town, University of Free-State, and University of the Witwatersrand. These services recognize the unique stressors and challenges that medical students face during their training. Counseling programs often provide support for academic stress, clinical experiences, burnout prevention, mental health concerns, and career guidance [9]. The counseling services include counseling sessions, peer support programs, and access to mental health resources and referrals.

The University of Cape Town’s second- and third-year MB ChB students participated in a study conducted in January 2017 as part of a contingency “mini-semester” course due to the postponement of classes and year-end exams in 2016 due to curriculum time being lost to the 2016 “Fees Must Fall” student protests [4]. The mini semester allowed students to move on to their next planned academic year after making up two months of lost instruction and learning time with six weeks of instruction and tests [4]. In light of the aforementioned contextual considerations and the ongoing national discourse around the mental health of junior physicians, clinicians, and academics, it is necessary to conduct a profile study of the students who will eventually fill these roles [4]. A total of 230 medical students were the subjects of an investigation by University of KwaZulu-Natal researchers on spirituality, depression, and quality of life [10]. By using the Zung Self Rating Scale, they were able to determine that 15.6% of students had scores that were suggestive of severe depression, with fourth-year students having the highest levels [10]. According to this local investigation, there were more mental health symptom loads during the clinical trial years. Longitudinal studies have demonstrated a 13.5% median absolute rise in depression symptoms following enrolment at medical school, according to the international literature on medical student mental health [10]. According to a different international mental health survey, MDD is the most frequent disorder with a lifetime prevalence of 21.2%, and it included first-year University of Cape Town students. With a lifetime frequency of 18.6%, generalized anxiety disorder was the second most prevalent disorder [3].

To bolster this perspective even further, research has shown that medical students are more likely than the general population to experience depression, anxiety, burnout, and general psychological discomfort [5,11,12,13,14,15,16]. This is probably due to the fact that during their training, medical students encounter a variety of organizational and personal challenges. Prior research has revealed that medical students encounter both patient distress and academically challenging learning situations [17,18]. Additionally, a number of studies carried out during the global coronavirus disease 2019 (COVID-19) pandemic revealed that medical students were a demographic at high risk for mental health issues [19].

In a similar vein, participants in a study at WITS University in South Africa said that weariness and stress were their two most common experiences [20]. Most individuals reported experiencing depersonalization and burnout coupled with emotional weariness [20]. There could be negative effects on one’s personal and professional life as a result of this high degree of burnout [21]. However, methylphenidate is used by 11% of undergraduate medical students at the University of Free State as a coping strategy and to improve performance, according to a recent survey conducted by Jain et al. [22,23]. The authors did point out that there is not much research that addresses the mental health issues that South African medical students experience. Therefore, this review highlights gaps. One significant gap revolves around the limited research focusing specifically on mental health issues among medical students in South Africa, with most studies being anecdotal or small-scale surveys. Additionally, there is a lack of standardized assessment tools and interventions tailored to the unique cultural and contextual factors influencing mental health among South African medical students. Therefore, the purpose of this study is to review the existing literature on the mental health needs and challenges faced by South African medical students. Also, this study seeks to identify gaps, trends, and areas for further research.

## 2. Literature Review Methodology

**Figure 1:**
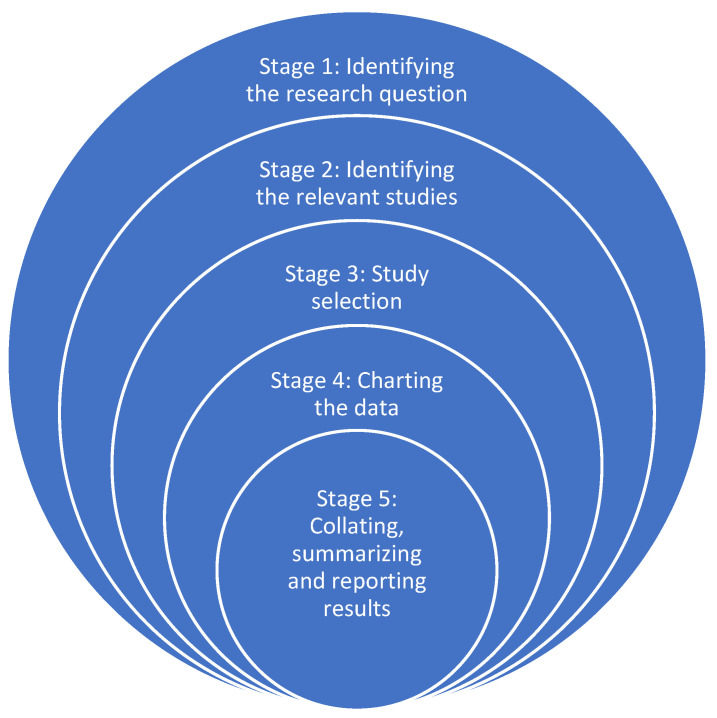
Five stages of scoping review proposed by Arksey and O’Malley [25].

A literature review is a survey of academic publications on a particular subject [24]. By giving a broad overview of current knowledge, it enables researchers to find pertinent theories, approaches, and gaps in the literature that they may use for their research articles [24]. The researchers opted for a scoping review approach, deeming it suitable for identifying knowledge gaps about the requirements and obstacles faced by medical students in South African academic institutions. The research adhered to Arksey and O’Malley’s five-stage scoping review proposal [25]. The five stages are outlined below in Figure 1.

### 2.1. Research Question

The following research questions guided this review:

- What are the prevalent mental health issues among medical students in South Africa?
- What are the contributing factors to mental health challenges among medical students in South African Universities?
- What are the existing support systems and interventions for addressing mental health concerns among medical students in South African universities?

### 2.2. Identifying Relevant Literature and Selection of Search Terms

**Figure 2:**
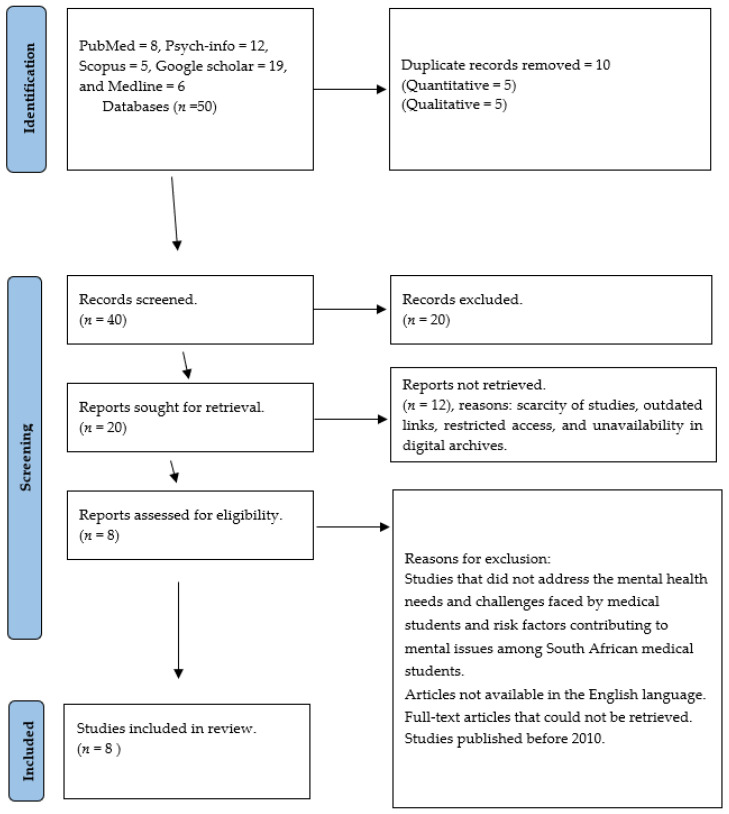
Flow chart representing the search strategy.

Boolean, logical, and keyword truncation processes were all incorporated into the search strategy. The primary language of reporting for the review is English. Electronic databases like PubMed, Psych-info, Scopus, Google Scholar, and Medline were searched for specific terms by the writers. In order to gather the required literature, Mendeley, grey literature, and significant academic websites were used as additional sources. The mechanism for searching was launched in December of 2023. The search terms were “Medical students” OR “Mental health”, OR “Challenges”, OR “South Africa”, OR “Universities” OR “Needs”, OR “Support systems”, OR “Mental health interventions” OR “Well-being”, OR “Anxiety, AND “Stress”. The authors retrieved 50 published studies. Only 12 studies were not retrieved because of the scarcity of studies specifically focusing on the mental health needs and challenges of medical students in South Africa, outdated links, restricted access, and unavailability in digital archives. Then, only 8 relevant studies meeting the inclusion criteria were included in this review. The selection process of the studies is shown in Figure 2 below.

#### 2.2.1. Inclusion Criteria

The inclusion criteria were studies published in peer-reviewed journals written in English between 2010 and 2023, studies that addressed the needs and mental health challenges faced by medical students in South Africa, and relevant studies that addressed the risk factors contributing to mental health issues among South African medical students and the available support systems and interventions.

#### 2.2.2. Exclusion Criteria

- Articles not available in the English language.
- Full-text articles that could not be retrieved.
- Studies published before 2010.
- Studies that did not address the mental health needs and challenges faced by medical students and risk factors contributing to mental issues among South African medical students.

#### 2.2.3. Quality Appraisal of the Study

To improve this study’s quality, two authors (L.W. and G.O.) performed a quality appraisal. To discuss the caliber of included studies, the authors were required to convene. Additionally, one reviewer was invited by the authors to evaluate and enhance the quality of this work. Using the Prisma Checklist 2020, which was modified for this study’s needs, the articles’ quality was assessed and analyzed. It looked at the articles’ robustness by assessing consistency, title clarity, abstract synthesis, state of the art, objectives, and method analysis for further research [26]. The checklist was modified to include 13 topics ((1) title; (2) abstract; (3) rationale; (4) objectives; (5) protocol; (6) eligibility criteria; (7) information sources; (8) inclusion/exclusion criteria; (9) data collection process; (10) study design; (11) main measure; (12) summary of main results; (13) conclusion and study limitations). The authors adopted a Likert scale of 0 (not reported/not specified), 1 (unclear/reported to some extent), and 2 (adequately reported) [27]. A score of 0 denotes that there is no information in the study about the subject at hand, whilst a score of 1 indicates that the researchers discussed the problem in passing without going into great detail. A score of two indicates that the researchers had a full discussion of the subject and provided a clear and intelligible explanation of the approach. The quality assessment of the studies that are part of this evaluation is displayed in Table S1 (Supplementary Material).

### 2.3. Data Extraction and Data Analysis

The authors (L.W. and G.O.) invited one reviewer to evaluate this work in order to reduce the possibility of bias. After that, they carefully examined each piece on their own. Together, the two writers and one reviewer examined fifty manuscripts to make sure there was consistency. Together, the reviewer and writers discussed data extraction and worked out any snags until they reached a consensus. The writers used Creswell’s Tesch approach in their thematic data analysis. For the eight included papers, the reviewer generated codes utilizing labels for various important aspects. By assessing the generated programs, the reviewer also searched for errors and duplications in the established themes. After that, the writers decided on the codes that were compared and merged to create the themes.

## 3. Results

The purpose of this scoping review was to examine the body of research on the needs and mental health difficulties seen by South African medical students, as well as the risk factors that may be involved in these problems. However, the authors were unable to conduct a thorough search due to the paucity of research available. In this review, only eight studies that were pertinent to the subject of the study were included. Using a thematic coding approach, the authors and reviewers determined the themes of this review. Three primary themes came out of this study: the frequency of mental illnesses, the risk factors that lead to poor mental health, and the interventions and support systems that universities have available.

### 3.1. Charting of Data

This review included eight studies. Most studies were quantitative, quantitative, and systematic reviews. It is very important to note that the authors focused more on published South African articles to review the literature on the needs and mental health challenges encountered by South African medical students. The characteristics of the eight studies included are tabulated in Table 1.

### 3.2. Collating, Summarizing, and Reporting the Results

The authors employed Cresswell’s Tesch method to formulate the themes.

#### 3.2.1. Theme 1: Prevalence of Mental Health Disorders

Studies reveal high rates of anxiety, despair, and burnout among South African medical students, raising serious concerns about mental health diseases within this population. Anxiety and depression were shown to be quite prevalent in South African studies. In total, 78% of medical students reported feeling stressed in a study by Naidoo et al. conducted in South Africa [28]. Women made up about 67% of the pupils who experienced stress [28]. According to a study by Van der Walt et al. conducted at UCT, roughly 45.9% of medical students had anxiety disorders and 36.4% had serious depressive disorders over the cutoff [4]. However, the majority of medical students were found to be distressed (97.06%) and extremely troubled (74.71%) in the study carried out by Dove et al. [23]. Comparably, a UKZN study revealed that a large percentage of medical students (15.6%) had evidence of severe depression symptoms, which are indicative of a probable mental disease. The study also revealed a high prevalence of depressive symptoms in the student population [10]. Analogous investigations carried out on South African medical students revealed that a high percentage (58.2%) of burnout was experienced [29,30,31]. It is important to highlight that there is a dearth of literature in South Africa about the mental health difficulties faced by medical students there. As a result, the writers are prepared to add to the body of South African literature on the subject.

#### 3.2.2. Theme 2: Risk Factors Contributing to Poor Mental Health

Poor mental health among medical students in South Africa is a multifaceted issue influenced by various factors. Firstly, the demanding academic environment places significant pressure on students, with a rigorous curriculum and high workload [31]. The constant need to excel academically, coupled with the competitive nature of medical education, can lead to stress, anxiety, and burnout [30,31]. Research has consistently shown that medical students experience higher levels of psychological distress compared to the general population, highlighting the impact of academic pressure on mental well-being [32]. Moreover, maintaining a healthy work–life balance becomes challenging for medical students. Juggling academic responsibilities with personal commitments can result in feelings of overwhelm and isolation. Studies conducted among healthcare students found that many medical students struggle to find time for self-care activities and relaxation due to the demands of their studies [33]. This imbalance can exacerbate mental health issues, contributing to emotional exhaustion and decreased overall well-being.

In addition to academic challenges, medical students often face financial stressors [28]. High tuition fees, living expenses, and the financial burden associated with pursuing a medical degree can take a toll on mental health. Financial constraints may force students to work additional hours or take out loans, adding to their stress levels [34]. Research suggests that financial difficulties are significantly associated with higher levels of psychological distress among medical students [35]. Furthermore, the lack of adequate support systems within academic institutions and in personal lives compounds mental problems [35].

#### 3.2.3. Theme 3: Available University Support Systems and Interventions

The purpose of university support systems and interventions for students’ mental health is multifaceted, aiming to enhance overall well-being, prevent mental health issues, reduce stigma, enhance academic success, build resilience, create a supportive community, foster personal growth, and address equity and inclusion.

##### Counseling Services

Counseling services in South African universities typically offer confidential support from trained professionals, such as psychologists or counselors, who specialize in addressing the unique challenges faced by medical students [36]. These services may include individual counseling sessions, group therapy, workshops, and crisis intervention. For example, the University of Cape Town provides counseling services through its student wellness services, offering short-term counseling, crisis intervention, and referrals to specialized mental health care when needed [4].

##### Peer Support Groups

Peer support groups are informal networks where medical students can connect with their peers to share experiences, provide mutual support, and offer practical advice. These groups may be organized by students themselves or facilitated by university staff. For instance, at the University of the Witwatersrand, the medical students’ council organizes peer support sessions where students can discuss academic stress, clinical experiences, and personal challenges in a supportive environment [31].

##### Mental Health Awareness Campaigns

South African universities often organize awareness campaigns and workshops to educate medical students about mental health issues, reduce stigma, and promote help-seeking behaviors [37]. These campaigns may include events such as mental health awareness weeks, guest lectures by mental health professionals, and interactive workshops on topics like stress management and resilience-building.

##### Access to Mental Health Professionals

South African universities often have access to mental health professionals, including psychologists, psychiatrists, and counselors, for students who require specialized support and treatment [37]. This may involve on-campus mental health clinics, referral networks with external providers, or partnerships with community mental health services.

## 4. Discussion

This scoping review delves into the intricate landscape of mental health needs and challenges among medical students within South African universities. The findings unearth three pivotal areas: the prevalence of mental health disorders, the risk factors contributing to poor mental health, and the available university support systems and interventions. Understanding and addressing these aspects are crucial for fostering a conducive environment for the well-being of medical students. The prevalence of mental health disorders among medical students in South African universities presents a concerning reality [4]. Our review identified a notable proportion of students grappling with various mental health issues. One of the significant gaps pertains to the lack of a comprehensive understanding of how historical, cultural, and institutional factors specific to South Africa impact the mental well-being of medical students. Additionally, there is a dearth of research focusing specifically on the effectiveness and accessibility of support systems and interventions tailored to address the mental health concerns of medical students in South Africa. By explicitly addressing these identified gaps in our manuscript, we aim to highlight the contributions of our review in advancing knowledge and informing future research and interventions in this area. This aligns with global trends, underscoring the universality of mental health challenges faced by medical students [36]. The demanding nature of medical education, coupled with the stressors inherent in clinical training, exacerbates susceptibility to mental health disorders [19]. To further contextualize these findings, it is imperative to explore the specific types of mental health disorders prevalent among medical students [19,27,28]. Research indicates a spectrum of conditions, including but not limited to anxiety, depression, burnout, and substance abuse [8,9,10]. These conditions not only compromise the well-being of medical students but also have profound implications for their academic performance, professional development, and overall quality of life.

Identifying the risk factors contributing to poor mental health among medical students is paramount for targeted intervention strategies. Our review unearthed multifaceted determinants exacerbating the vulnerability of medical students to mental health disorders. These encompass both academic and non-academic stressors, spanning from rigorous academic demands, high workload, and intense competition to social isolation, financial pressures, and inadequate support systems [20,25]. Furthermore, societal and cultural factors intersect with the unique challenges faced by medical students in South Africa [28]. The prevailing stigma surrounding mental health within the medical community, coupled with cultural norms emphasizing resilience, often hinder help-seeking behaviors among students [37]. Additionally, systemic issues such as inequities in access to mental health resources and discriminatory practices perpetuate disparities in mental health outcomes among medical students from diverse backgrounds [13,14,15]. For instance, in terms of discriminatory practices, medical students who are members of the LGBTQ+ community face discrimination related to their sexual orientation or gender identity, contributing to feelings of marginalization and stress. Additionally, medical students from lower socioeconomic backgrounds may experience discrimination based on their financial status, leading to a sense of inadequacy [25].

In navigating the complex landscape of mental health among medical students, it is imperative to assess the efficacy and accessibility of existing university support systems and interventions [33]. Our review sheds light on the diverse array of resources and initiatives implemented by South African universities to address mental health concerns among medical students. These support systems encompass a spectrum of interventions, ranging from preventive measures such as stress management workshops, mental health awareness campaigns, and peer support networks to reactive strategies including counseling services, psychiatric consultations, and crisis intervention protocols. Additionally, academic accommodations and flexible scheduling options are instrumental in mitigating the academic burden on students experiencing mental health challenges [33,34,35]. However, despite the availability of these resources, significant barriers persist in accessing mental health support within South African universities [38]. These barriers encompass logistical constraints, inadequate funding, understaffing of mental health professionals, and persistent stigma surrounding mental illness.

This study can be strengthened by the biopsychosocial model of health. This model emphasizes that health outcomes are influenced not only by biological factors but also by psychological aspects, as well as social determinants [39]. By acknowledging the complex interaction between these dimensions, the biopsychosocial model guides research and intervention efforts to address health challenges comprehensively, recognizing that individual well-being is shaped by a multitude of interconnected factors spanning biological, psychological, and social realms.

## 5. Study Limitations

Firstly, the inclusion criteria may have led to the omission of relevant studies published in languages other than English, potentially limiting the comprehensiveness of this review. The study focused more on South African studies that addressed the mental health needs and challenges encountered by South African medical students. It is possible that our search strategy missed relevant studies. The heterogeneity of study methodologies across the included literature may introduce variability and complexity in synthesizing the results. There is a lack of studies focusing on the mental health needs and challenges among medical students in South Africa, which affects the quality and reliability of this study. Notable, the studies included were cross-sectional, omitting other research methodologies.

## 6. Conclusions and Recommendations

In conclusion, this scoping review underscores the pressing need for comprehensive mental health support programs within South African medical education institutions to address the prevalent challenges faced by medical students. Recommendations include implementing proactive measures to reduce academic pressure, providing accessible mental health services and support systems, destigmatizing discussions around mental health, and promoting self-care practices among students. Furthermore, fostering a culture of openness and support within medical schools, along with faculty training on recognizing and addressing mental health concerns, is essential. Collaborative efforts involving students, faculty, administrators, and mental health professionals are necessary to create a supportive environment conducive to the well-being of medical students in South Africa. Additionally, further research is warranted to evaluate the effectiveness of interventions and strategies aimed at promoting mental health resilience among medical students.

## Figures and Tables

**Table 1:** Characteristics of the studies.

Author/Year	Study Objectives	Study Design	Sample Size (*n*)	Outcomes
Van der Walt et al. (2020) [4].	To determine the rates of depression and anxiety among medical students and to examine the associations with various sociodemographic variables.	Cross-sectional study.	473	High prevalence of major depressive disorder and anxiety.
Pillay et al. (2016) [10].	To determine the prevalence of moderate and severe depressive symptoms in this population and explore potential correlations between spirituality, depression, and quality of life.	Quantitative study.	230	There was a high prevalence of depressive symptoms in the medical students.
Dove et al. (2023) [21].	To evaluate the well-being of fourth-year students in the Bachelor of Medicine and Surgery (MBBCh) degree at the University of the Witwatersrand (WITS).	Cross-sectional study.	333	Most participants were distressed.
Jain et al. (2017) [22].	To investigate the prevalence of the non-medical use of methylphenidate and knowledge of this drug among undergraduate medical students at the University of the Free State.	Cross-sectional study.	541	Methylphenidate users’ median knowledge was greater than non-users’, and methylphenidate knowledge increased from first-year and second-year students to third-year to fifth-year students.
Jain et al. (2018) [23].	To investigate the prevalence of cannabis use among undergraduate medical students at the University of the Free State (UFS), and the extent of their knowledge about the substance.	Cross-sectional study.	541	The median knowledge score of students who used cannabis tended to be higher than that of students who did not use cannabis.
Naidoo et al. (2014) [28].	To explore the prevalence and causes of stress, its impact on students, and their coping strategies in a racially diverse cohort of final-year medical students exposed to a problem-based learning curriculum in South Africa.	Cross-sectional study.	2008	Academic and personal problems were the main sources of stress.
Colby et al. (2018) [29].	To determine the association between the levels of burnout and quality of life among fourth-year medical students at the University of the Free State (UFS).	Quantitative.	91	High levels of reported burnout.
Van der Merwe et al. (2020) [30].	To investigate burnout and associated factors among undergraduate students at a South African medical school.	Cross-sectional study.	500	High scores on the subscale reflect low levels of burnout in related areas.

## Data Availability

No new data were created or analyzed in this study. Data sharing is not applicable to this research.

## Supplementary Materials

The following supporting information can be downloaded at: https://www.mdpi.com/article/10.3390/ijerph21050593/s1, Table S1: Quality Appraisal of the studies (tool adopted from Gomes et al., 2023) [27].
