# Supplementary material for: A Scoping Review of Mental Health Needs and Challenges among Medical Students within South African Universities
Source: Int J Environ Res Public Health. 2024 May 4;21(5):593. doi: 10.3390/ijerph21050593 (PMC11120686; doi:10.3390/ijerph21050593)
Supplement: Supplementary file 1 [file ijerph-2979403-supplementary.pdf › ijerph-2979403-supplementary.pdf]

Table S1: Quality Appraisal of the studies (Tool adopted from Gomes et al 2023) [27].

Articles	1	2	3	4	5	6	7	8	9	10	11	12	13	Total
Van der Walt et al. (2020) [4].	2	2	2	2	2	2	2	2	2	2	2	2	2	26 High quality
Pillay et al. (2016) [10].	2	2	2	2	2	2	2	2	2	2	2	2	2	26 High quality
Dove et al. (2023) [21].	2	2	2	2	2	2	2	2	2	2	2	2	2	26 High quality
Jain et al. (2017) [22].	2	2	2	2	2	2	2	2	2	2	2	2	2	26 High quality
Jain et al. (2018) [23].	2	2	2	2	2	2	2	2	2	2	2	2	2	26 High quality
Naidoo et al. (2014) 28].	2	2	2	2	2	2	2	2	2	2	2	2	2	26 High quality
Colby et al. (2018) [29].	2	2	2	2	2	2	2	2	2	2	2	2	2	26 High quality
Van der Merwe et al. (2020) [30].	2	2	2	2	2	2	2	2	2	2	2	2	2	26 High quality
